# Understanding invasion history and predicting invasive niches using genetic sequencing technology in Australia: case studies from Cucurbitaceae and Boraginaceae

**DOI:** 10.1093/conphys/cow030

**Published:** 2016-08-26

**Authors:** Razia S. Shaik, Xiaocheng Zhu, David R. Clements, Leslie A. Weston

**Affiliations:** 1 Graham Centre for Agricultural Innovation (Charles Sturt University and NSW Department of Primary Industries), Locked Bag 588, Wagga Wagga, NSW 2678, Australia; 2 Department of Biology, Trinity Western University, Langley, BC, CanadaV2Y 1Y1

**Keywords:** DNA barcoding, genetic profile, invasion history, plant invasion, sequencing technology

## Abstract

Part of the challenge in dealing with invasive plant species is that they seldom represent a uniform, static entity. Often, an accurate understanding of the history of plant introduction and knowledge of the real levels of genetic diversity present in species and populations of importance is lacking. Currently, the role of genetic diversity in promoting the successful establishment of invasive plants is not well defined. Genetic profiling of invasive plants should enhance our understanding of the dynamics of colonization in the invaded range. Recent advances in DNA sequencing technology have greatly facilitated the rapid and complete assessment of plant population genetics. Here, we apply our current understanding of the genetics and ecophysiology of plant invasions to recent work on Australian plant invaders from the Cucurbitaceae and Boraginaceae. The Cucurbitaceae study showed that both prickly paddy melon (*Cucumis myriocarpus*) and camel melon (*Citrullus lanatus*) were represented by only a single genotype in Australia, implying that each was probably introduced as a single introduction event. In contrast, a third invasive melon, *Citrullus colocynthis*, possessed a moderate level of genetic diversity in Australia and was potentially introduced to the continent at least twice. The Boraginaceae study demonstrated the value of comparing two similar congeneric species; one, *Echium plantagineum*, is highly invasive and genetically diverse, whereas the other, *Echium vulgare*, exhibits less genetic diversity and occupies a more limited ecological niche. Sequence analysis provided precise identification of invasive plant species, as well as information on genetic diversity and phylogeographic history. Improved sequencing technologies will continue to allow greater resolution of genetic relationships among invasive plant populations, thereby potentially improving our ability to predict the impact of these relationships upon future spread and better manage invaders possessing potentially diverse biotypes and exhibiting diverse breeding systems, life histories and invasion histories.

## Abstract (precis)

The development of genetic profiles of invading plants can provide insight towards a better understanding of niche utilization. Recent advances in sequencing techniques have greatly facilitated genetic assessment and profiling. Here we review our recent studies on selected Australian Cucurbitaceae and Boraginaceae to demonstrate the application of genetic sequencing across a selection of invasive Australian plants.

## Introduction

The seriousness of the global challenge due to invasive plant species has been increasingly recognized in the past two decades, while climate change and increased global trade have served to accelerate plant invasion (Beerling *et al*., 1995; Bunce and Ziska, 2000; Ding *et al*., 2008; McDonald *et al*., 2009; Clements and Ditommaso, 2011; Hyvönen *et al*., 2012; Gallagher *et al*., 2013; Singh *et al*., 2013; Clements *et al*., 2014; Seebens *et al*., 2015). In Australia, >3000 non-native plant species are now recorded as naturalized (Stow *et al*., 2014), and threats from these species are increasing exponentially. Many of these invaders have become noxious or weedy, with an estimated annual cost of over 4 billion AUD (Olson, 2006). For example, one invasive weed, bitou bush (*Chrysanthemoides monilifera*), is associated with population decline in 63 rare and threatened native plant species in New South Wales alone (Kohli *et al*., 2008). In addition, when present in agricultural lands, weed infestation contributes to the majority (34%) of total losses attributable to pests relative to all crop pests (Oerke, 2006).

In order to address the challenges associated with invasive weeds, systems of prediction are being developed, in terms of both associated theoretical frameworks that attempt to identify the major predictors of invasion (e.g. Daehler, 2003) and models that predict the extent of invasion. In particular models, potential regions of further invasion are identified by evaluating current home ranges and predicted ranges where species may invade based on climate change and other factors (e.g. Kriticos *et al*., 2005; Ebeling *et al*., 2008; McDonald *et al*., 2009). Many attempts have been made to predict the scope of future invasions, but information on critical aspects of invasive plant biology is often lacking, including the ability of species to evolve in response to selection pressures, such as climate change (Clements and DiTommaso, 2011, 2012).

Fundamental to the nature of a given species or individual organism is a plant's genetic identity. Gene regulation and environmental interactions determine the physiological nature of a plant as it develops from seeds and/or other types of propagules, which in turn determines its eco-physiological success and eventual impact on ecosystems and/or human economies. A common shortcoming in the management of invasive plants is the failure to recognize a given weed species as not only a single genetic identity but a collection of populations that may vary greatly across a variety of scales from local to regional to global (Meekins *et al*., 2001; Lavergne and Molofsky, 2007; Prentis *et al*., 2008). Therefore, although a species may be a single entity by definition, populations of a particular species may exhibit both genotypic and phenotypic variation. Thus, their successful management may be improved greatly by addressing specific genetic manifestations of the species resulting in phenotypic variation attributable to genetic variation and/or plasticity.

Genotypic and phenotypic diversity is also observed in invaders across all taxa, but it is important to highlight particular features of plants that are crucial in predicting the success of plant invasions. Plant breeding systems and life histories are therefore key considerations. In terms of breeding systems, plants may fall anywhere on a spectrum from obligate outcrossing to 100% selfing (Clements *et al*., 2004). Many plants forgo reproduction by seed as well, often making use of the advantages afforded by vegetative propagation from already vigorously growing plant parts. In terms of plant life histories, whether a plant is an annual or perennial or some intermediate of the two extremes can influence whether or not it is or could become a problematic invader (Meekins *et al*., 2001; Lavergne and Molofsky, 2007; Prentis *et al*., 2008).

Although we know much about the genetics of particular invasive species, there are still many gaps in our knowledge (Bock *et al*., 2015). For example, there remain important questions around what factors influence the primary sources of genetic variation, the role of genetic bottlenecks in potentially hindering the success of plants at the fringe of an invasion wave, and whether propagule load is more important than genetic diversity in promoting establishment; these are questions that may be answered by both genomic studies and studies of plant ecophysiology using model organisms (Bock *et al*., 2015).

In this review, we compare and contrast the genetic diversity of two models; Australian congeneric invaders representing the Cucurbitaceae and Boraginaceae. The Boraginaceae model compares two congeric invaders introduced to Australia in a similar time frame; one highly successful invader and the other a niche colonizer with similar morphological, chemical and biological features (Skoneczny *et al*., 2015; Zhu X, Skoneczny D, Gopurenko D, Meyer L, Lepschi BJ, Weston PA, Callaway RM, Gurr GM, Weston LA. A tale of two plant invaders: comparison of the ecology and genetics of *Echium plantagineum* and *E. vulgare* in southern Australia. *Scientific Reports*, under review; Zhu *et al*., 2016). The Cucurbitaceae model compares three related melons that appear to have been introduced to Australia via camel trading routes established in the 1800s, with genetic diversity among the three largely selfing species varying from existing as a single in genotype in Australia for prickly paddy melon (*Cucumis myriocarpus*) and camel melon (*Citrullus lanatus*) to the more heterogeneous populations of *Citrullus colocynthis* composed of two major introduced genotypes in Australia (Shaik *et al*., 2015). Two of the melon species (*C. myriocarpus*) and (*C. lanatus*) are annuals, whereas *C. colocynthis* is perennial. The experience of working with these two different plant families in Australia using similar genetic analysis methods enables us to draw some general conclusions on the value of such analyses in characterizing continental invasions by a variety of taxa. Thus, our overall objective is to examine how recent advancements in genetic characterization and sequence analysis can be applied successfully to invasive plants with varying life histories, breeding systems and invasion histories.

## Recent advances in DNA sequencing for invasive plants

One key innovation in recent years is the development of DNA barcoding for rapidly characterizing invasive plant genetics. DNA barcoding can be defined as ‘a diagnostic technique in which short DNA sequence(s) can be used for species identification’ (Savolainen *et al*., 2005). DNA barcoding using the 648 bp region of the mitochondrial gene cytochrome *c* oxidase I is a well-accepted method of species identification in animals (Wiemers and Fiedler, 2007; Lahaye *et al*., 2008; Hollingsworth *et al*., 2009). Successful use of barcoding requires that genetic distance between species is larger than within-species distance. Its success also depends on monophyly of the species examined (Wiemers and Fiedler, 2007). Interestingly, species boundaries in plants are typically less pronounced than in animals. In some cases, up to 50% of plants show higher levels of gene tree paraphyly, and interspecific hybridization exacerbates this, often making fine-scale species distinction within plants difficult (Fazekas *et al*., 2009). Owing to the absence of a standard barcode region in plants, appropriate sampling and a careful choice of markers are essential prerequisites for correct plant species identification (Mort *et al*., 2007; Fazekas *et al*., 2009).

The Consortium for the Barcode of Life (CBOL) recommended a two-marker-based system as a barcode for flowering plants, i.e. maturase K (*mat*K) and ribulose-bisphosphate carboxylase gene (rbcL; Hollingsworth *et al*., 2009). Although this combination of gene regions works for some plants (Steven and Subramanyam, 2009), it may not be useful in others (Zhang *et al*., 2014). This failure can be attributed to low sequence polymorphism between species at rbcL and difficulty in sequence retrieval in the case of *mat*K, for example, as seen in Zingiberaceae (Kress *et al*., 2005). Some studies have suggested that the *mat*K region alone can potentially be used for plant barcoding, e.g. for species distinction in *Annona*, a genus belonging to pawpaw/sugar apple family Annonaceae (Lahaye *et al*., 2008; Hollingsworth *et al*., 2009; Larranaga and Hormaza, 2015). Molecular systematics and phylogeographic studies have also extensively used evolutionarily conserved chloroplast DNA (Parducci and Szmidt, 1999; Desplanque *et al*., 2000; Xu *et al*., 2001). The chloroplast genes, although uniparentally inherited and highly conserved, can be extremely useful for species and haplotype distinction in some cases. In *Dendrobium* species, 100% species resolution was observed by using the chloroplast *psb*A-*trn*H intergenic spacer (Yao *et al*., 2009).

For plants in the genus *Citrullus*, genetic diversity has also been determined by using chloroplast DNA and sequencing analysis of several non-coding regions (Dane and Bakhtiyarova, 2003; Dane *et al*., 2004; Dane and Liu, 2007). Relative to nuclear markers, maternally inherited chloroplast markers may sometimes be associated with low polymorphism, caused by slow evolution owing to a reduced rate of substitution at synonymous sites and also in non-coding inverted repeat sequences (Wolfe *et al*., 1987). Furthermore, chloroplast capture events and intraspecific hybridizations may cause selective sweeps, resulting in shared haplotype formation and incongruent gene trees, as noted in Australian populations of golden wattle (*Acacia pycnantha*; Ndlovu *et al*., 2013). This, in turn, can lead to failure in species identification when using a combined data set of multiple chloroplast genes, as was observed in some cases (Rosenthal *et al*., 2008; Twyford, 2014), such as willow (*Salix* spp.; Percy *et al*., 2014) and sweet chestnut fruit (*Castanea* spp.; Li and Dane, 2013).

The evolution of nuclear genes is independent from plastid DNA; therefore, nuclear regions, including the internal transcribed spacer region (ITS), may also be required for increased resolution (Chase *et al*., 2005) and hybridization testing (Chase *et al*., 2005; Zhang *et al*., 2013). The ITS from nuclear ribosomal DNA typically shows greater discriminatory power (Hollingsworth *et al*., 2011) and is easily amplified by using universal primers in some plant molecular studies. It has been successfully used for phylogenetic studies in some families, e.g. in the Euphorbiaceae (Pang *et al*., 2010). The ITS region was also used to infer phylogenetic relationships in *Cucumis* and *Citrullus* (Jarret and Newman, 2000; Garcia-Mas *et al*., 2004). Some limitations of ITS use include difficulty in obtaining the sequences and incomplete concerted evolution of the gene, leading to divergent paralogous copies within the same individual. Additionally, polymorphic sites need to be scored carefully (Mort *et al*., 2007; Hollingsworth *et al*., 2011).

Systematists have argued that dependence on a single sequenced region may result in a distorted picture of phylogenetic relationships, as incongruence has been observed between phylogenetic trees of nuclear and chloroplast origin (Fehrer *et al*., 2007); hence, phylogenetic inferences are now being made using multiple gene regions (Soltis and Soltis, 2004). Some researchers recommend using multiple markers from independent genomes, including a chloroplast and a nuclear gene together, for better taxon discrimination (Kress *et al*., 2005; Mort *et al*., 2007; Zhang *et al*., 2014). This helps to overcome the inherent inaccuracies of using single gene markers (Rubinoff *et al*., 2006; Mort *et al*., 2007). A combination of nuclear *G3pdh* and chloroplast *ycf6-psb*M regions was successfully used to distinguish species within *Citrullus* (Dane *et al*., 2007). This suggests that successful identification mainly depends on successful determination of a gene region or a combination of gene regions.

Numerous markers have also been used for plant genetic diversity and species identification studies during the last few decades, including Simple Sequence Repeat (SSR), Amplified Fragment Length Polymorphism (AFLP), Restriction Fragment Length Polymorphism (RFLP), Random Amplified Polymorphic DNA (RAPD) and Inter Simple Sequence Repeat (ISSR). Compared with DNA barcoding, these markers can be cheaper and sometimes more polymorphic. However, they can also be impracticable because of erroneous results in scoring (electropherogram base calling; Devey *et al*., 2009). Successful use of marker-based systems for analysis of diversity requires subjective human judgment and editing, which can sometimes be overcome using PeakScanner and SPAGeDi software (Ley and Hardy, 2013). In addition, markers such as RAPDs and ISSRs are generally subject to reproducibility issues between laboratories (Gaskin *et al*., 2011). The cost of sequencing technology and analysis has recently been dramatically reduced. Complete plastid genome sequencing or even whole genome sequencing using next generation sequencing may eventually prove affordable, and these technologies will provide much useful information for those willing to work with large data sets and perform bioinformatics (Su *et al*., 2011).

Recently, we conducted DNA sequence analysis on five Australian invasive plant species in two families, the Cucurbitaceae and Boraginaceae. These species exhibit a variety of breeding systems, life histories and introduction histories across Australia (Table 1).

**Table 1:** Comparison of the plants featured in DNA sequencing case studies of invasive species from Cucurbitaceae (data from Shaik *et al*., 2015) and Boraginaceae (data from Zhu *et al*., 2016) in Australia

Plant taxa	Life cycle	Chloroplast haplotypes	Nuclear genotypes	Invasiveness in Australia	Breeding system in Australia
Cucurbitaceae					
*Cucumis myriocarpus*	Annual	1	1	H	SC
*Citrullus lanatus*	Annual	1	1	H	SC
*Citrullus colocynthis*	Perennial	2	4	H	SC
Boraginaceae					
*Echium plantagineum*	Annual	12	2	H	SC
*Echium vulgare*	Perennial	2	4	L	SC

Invasiveness ratings: H, high; L, low. Breeding systems: SC, self-compatible; SI, self-incompatible.

## Cucurbitaceae case study

### Species profiles

**Figure 1:**
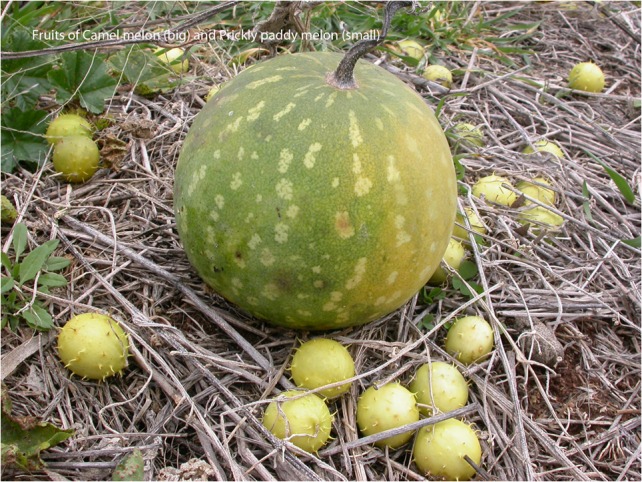
Fruits of invasive Cucurbitaceae in Australia. The large fruit is *Citrullus lanatus* (camel melon), which has an average diameter of 7–10 cm. The smaller fruit is *Cucumis myriocarpus* (prickly paddy melon), which has an average diameter of 2–3 cm. The fruit of *Citullus colocynthis* (colocynth melon) is similar in size and appearance to that of *C. lanatus*, but *C. colocynthis* rind tends to have a mottled or mosaic pattern as opposed to the spotted or striped pattern seen on *C. lanatus* (Shaik *et al*., 2012).

Three cucurbitaceous invasive melons, camel melon [*Citrullus lanatus* (Thunb.) Matsum. and Nakai], prickly paddy melon (*Cucumis myriocarpus* L.) and colocynth melon [*Citrullus colocynthis* (L.) Schrad.] are currently distributed across Australia (see Fig. 1 for illustrations of the first two), invading crops, fallow lands and natural habitats (Leys *et al*., 1990; Parsons and Cuthbertson, 2001; Johnson *et al*., 2006b; Richardson *et al*., 2006). In Australia, wild melons were cited as one of the main summer fallow weed problems in a Grains Research & Development Corporation (GRDC) survey conducted in 2014 (Llewellyn *et al*., 2016). Their expansion is likely to continue unless adequate control strategies are implemented. The first two are annual vines that germinate during spring, fruit during summer and senesce during autumn (Parsons and Cuthbertson, 2001), whereas the third, colocynth, is a perennial vine. Australian summer weeds can result in up to 1 ton wheat yield loss per hectare if left uncontrolled, causing a loss of soil moisture of up to 50 mm that would otherwise have been useful for subsequent winter crops (Van Rees *et al*., 2011).

There has been confusion regarding identification of wild melons in Australia before flowering, at both the morphological and the taxonomic level. Herbicide control at the seedling stage is now recommended (Johnson *et al*., 2006a). However, clear identification of the cucurbitaceous species in question is challenging, as some herbicides do not fully control all three species (Johnson *et al*., 2006a). Wild melons ascribed to the species *C. lanatus* are also a prominent weed in other countries, including New Zealand and the USA (Parsons and Cuthbertson, 2001; Futch and Hall, 2003; Grichar *et al*., 2010; Abd El-Ghani *et al*., 2011). The other annual wild melon most common in Australia, *C. myriocarpus*, has also become naturalized in southern Europe and California (Grubben and Denton, 2004). In addition, the perennial wild melon species, *C. colocynthis*, is a weed in Australia and parts of Asia (Parsons and Cuthbertson, 2001; Dane *et al*., 2007; Burrows and Shaik, 2014).

### DNA sequencing study insights

The two annual wild melon species share similar vegetative growth and produce yellow flowers; therefore, they are often confused. This is particularly so before fruit formation in the case of *C. myriocarpus*. The perennial species, *C. colocynthis*, is closely related, shares morphological similarity with *C. lanatus* and is often misidentified even on fruit formation. Interestingly, initial trials with chloroplastidic *mat*K gene did not result in separation of these two congeneric species because their sequences were 100% similar (Shaik *et al*., 2011). However, a chloroplast gene (*ycf*6-*psb*M) and a nuclear gene (*G3pdh* intron region) based on Dane *et al*. (2007) proved useful in evaluating the interspecific and intraspecific variability among the three cucurbitaceous invasive species.

The results of extensive sampling across Australia showed that *C. lanatus* and *C. myriocarpus* were each represented by a single genotype and haplotype, indicating that the populations present were derived from a single introduction event or multiple introduction events of a single genotype (and subsequently selfing). Moderate levels of genetic diversity were present among Australian *C. colocynthis*, and this species sorted geographically into separate haplotypes found in eastern and western regions, suggesting at least two separate introductions from two different source populations (Shaik *et al*., 2015). These findings suggested that the two gene regions described above can be used to identify the invasives in question as *C. myriocarpus* subsp. *myriocarpus* for Australian prickly paddy melon and *C. lanatus* var. *citroides* for camel melon, previously described in the literature as the Australian wild melon *C. lanatus* var*. lanatus* (Shaik *et al*., 2011, 2012, 2015).

The findings of Shaik *et al*. (2015) suggest that an integrative approach, using both morphological characters and DNA-based methods, including sequence analysis for identification, is likely to be more successful than either approach alone. Based on the discovery that *C. lanatus* is a single genetic entity, it is likely that the Australian population can be controlled effectively by one efficacious method of control, barring any local variations in management required as a result of phenotypic differences. This is also thought to be the case with *C. myriocarpus*. However, populations of *C. colocynthis* may require differential methods of management should genotypic and phenotypic differences predominate among eastern and western populations.

Other well-described hypotheses that will not be discussed in detail in this review provide explanations of how populations with low genetic diversity can become invasive and include pre-adaptation (Dlugosch and Parker, 2007; Clark *et al*., 2013; Dostál *et al*., 2013), phenotypic plasticity and enhanced resource availability (Callaway and Aschehoug, 2000; Graebner *et al*., 2012; Stricker and Stiling, 2013), natural enemy release (Hinz *et al*., 2012) or a combination of factors (Geng *et al*., 2007; Eriksen *et al*., 2012; Vergeer and Kunin, 2013). Additional studies on the roles of breeding system and pollinator interactions may shed light on these successfully inbreeding invasive plants.

## Boraginaceae case study

### Species profiles

**Figure 2:**
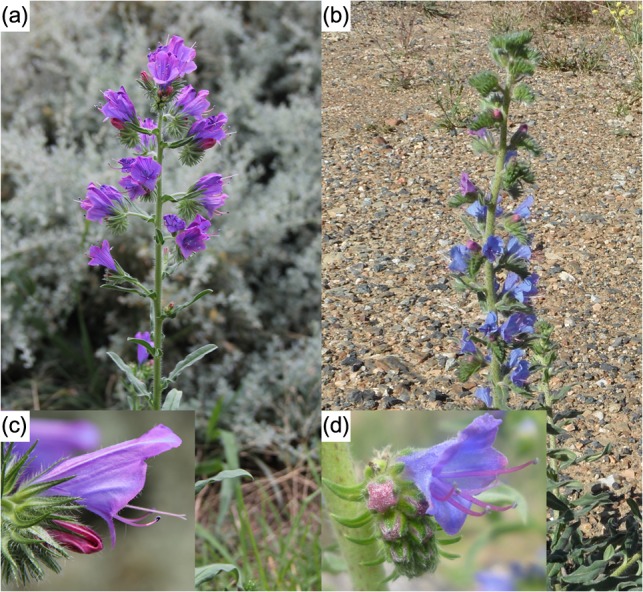
(**a**) Inflorescence of *Echium plantagineum* (Paterson's curse). (**b**) Inflorescence of *Echium vulgare* (viper's bugloss). (**c** and **d**) Note that flower size in *E. plantagineum* (**c**) is typically larger and exhibits two protruding stamens, in contrast to smaller flower size in *E. vulgare* with a lack of protruding stamens (**d**).

Australia has two exotic invasive *Echium* species: Paterson's curse (*E. plantagineum*; Fig. 2a) and viper's bugloss (*E. vulgare*; Fig. 2b). Both species originated in southern Europe and were introduced to Australia during the 19th century (Piggin, 1982; Klemow *et al*., 2002). The former soon became a serious weed after introduction, covering almost all biogeographical regions in southern Australia. Today, it is estimated to infest >33 million hectares, causing >250 million AUD in losses to the meat and wool industries (NRM South and the Southern Tasmanian Councils Authority, 2016). In contrast, *E. vulgare*, although more common across Europe, is a niche colonizer in Australia and is currently found in only a small subset of biogeographical regions across New South Wales, Victoria and Tasmania.

Correct identification of these congeric *Echium* species has typically caused confusion in Australia, especially before anthesis. Prior to the 1950s, the common name ‘Paterson's curse’ was used for both species (Parsons, 1973). Piggin (1977) reported misidentification between the two in Australian herbaria collections, which contributes to confusion in tracking the dynamics of dispersal over time. The Australian introduction history of the highly invasive *E. plantagineum* is also not clear. Piggin (1982) suggested that *E. plantagineum* was introduced as an ornamental species from England; however, it is more likely that this species was introduced to Australia from Spain, potentially via South Africa, as a seed contaminant of hay through importation of Merino sheep in the late 18th century (Zhu *et al*., 2014b).

### DNA sequencing study insights

The genetic diversity of *E. plantagineum* and *E. vulgare* was evaluated by sample collection from Queensland, New South Wales, Australian Capital Territory, Victoria, South Australia, Northern Territory and Western Australia. Results indicated that both *Echium* species were routinely identified and separated using any of four DNA regions under evaluation, which included one nuclear region ITS and three chloroplast regions (*trn*L intron, *trn*L-*trn*F spacer and *psb*A-*trn*H spacer; Zhu *et al*., 2014a). *Echium plantagineum* and *E. vulgare* possessed 12 and two haplotypes each, respectively, when separated using three chloroplast regions (Zhu X, Skoneczny D, Gopurenko D, Meyer L, Lepschi BJ, Weston PA, Callaway RM, Gurr GM, Weston LA. A tale of two plant invaders: comparison of the ecology and genetics of *Echium plantagineum* and *E. vulgare* in southern Australia. *Scientific Reports*, under review). The more successful invader, *E. plantagineum*, showed significantly higher levels of genetic diversity than did the less invasive *E. vulgare*, which supports the hypothesis that a certain level of genetic diversity is associated with success of invasion in herbaceous plants (Jose *et al*., 2013).

The relative pattern of introduction of Australian *E. plantagineum* was also observed through sequence analysis experimentation. The introduction of *E. plantagineum* was first reported historically in Albury (southern New South Wales), Gladstone (South Australia) and Western Australia in 1880, 1889 and 1881, respectively (Piggin, 1977; Kloot, 1982). Spatial-specific haplotypes were found near these sites, while western New South Wales, a buffer area between the South Australia and New South Wales introduction events, showed the greatest number of haplotypes detected in the study (Zhu X, Skoneczny D, Gopurenko D, Meyer L, Lepschi BJ, Weston PA, Callaway RM, Gurr GM, Weston LA. A tale of two plant invaders: comparison of the ecology and genetics of *Echium plantagineum* and *E. vulgare* in southern Australia. *Scientific Reports*, under review). These findings support the hypothesis that multiple introductions of *E. plantagineum* occurred across Australia. However, to unravel the pathway of *E. plantagineum* introduction to Australia further, additional investigation is required and is currently ongoing through evaluation of a global collection of samples.

**Figure 3:**
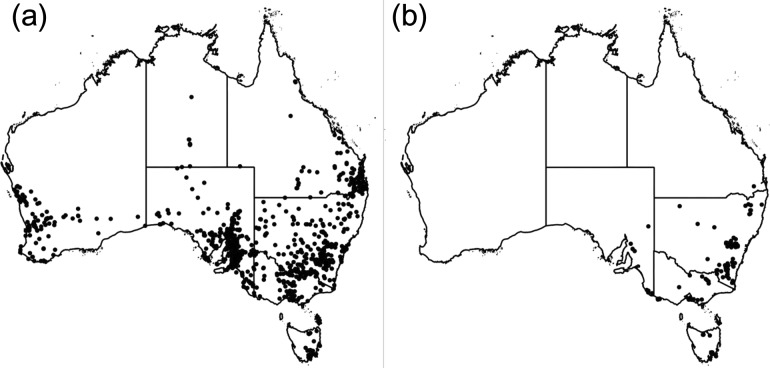
Distribution of *Echium plantagineum* (Paterson's curse; **a**) and *Echium vulgare* (viper's bugloss; **b**) in Australia. Source of distribution data: Australia's Virtual Herbarium, 2015.

This study also highlights the limited genetic diversity found in Australian specimens of *E. vulgare* (Zhu X, Skoneczny D, Gopurenko D, Meyer L, Lepschi BJ, Weston PA, Callaway RM, Gurr GM, Weston LA. A tale of two plant invaders: comparison of the ecology and genetics of *Echium plantagineum* and *E. vulgare* in southern Australia. *Scientific Reports*, under review)*. Echium vulgare* is restricted in its spread across Australia and is mainly found in the southeastern highlands (Fig. 3). As a perennial, it requires vernalization to induce flowering (Klemow *et al*., 2002), and is less drought tolerant when compared with *E. plantagineum*. Currently, *E. vulgare* is potentially under threat owing to its limited habitat as a niche colonizer. With exposure to a changing climate, its range may be further restricted in future years. In contrast, *E. plantagineum*, while already much more widely distributed than *E. vulgare* in Australia (Fig. 3), is predicted to become more invasive given its ability to withstand drought and its relatively high levels of genetic diversity, which may allow it successfully to adapt to recently changing environmental conditions across southern Australian biogeographical regions (Zhu X, Skoneczny D, Gopurenko D, Meyer L, Lepschi BJ, Weston PA, Callaway RM, Gurr GM, Weston LA. A tale of two plant invaders: comparison of the ecology and genetics of *Echium plantagineum* and *E. vulgare* in southern Australia. *Scientific Reports*, under review).

## Value of DNA sequencing for a diverse array of invasive plants

### Diverse breeding systems

Modes of reproduction and dispersal play a vital role in determining the genetic structure of a population of a particular species (Barrett *et al*., 2008; Petanidou *et al*., 2012). Many factors influence the genetic diversity of populations (Loveless and Hamrick, 1984), including ecological parameters. Genetic variation among populations is often solely dependent on the breeding system of the species (Schoen and Brown, 1991). Within-population genetic diversity is often reported to be low in the case of inbreeding populations and high in the case of outcrossing populations (Charlesworth and Charlesworth, 1995). In comparison, the among-population diversity was high (up to 51% of the total genetic diversity) in selfing and endemically distributed species. This is in direct contrast to outcrossing populations that were widely distributed geographically or those that were wind dispersed, where only a small proportion (~10%) of total genetic diversity was observed among populations (Hamrick *et al*., 1990, 1992; Hooper and Haufler, 1997).

For the Australian weeds discussed above, genetic characterization provided helpful insights into invasion history, although breeding systems varied widely in each case. *Citrullus lanatus* and *C. myriocarpus* reproduce by selfing and form large infestations of a single haplotype in the invaded range. The breeding system in *Echium* species features protandrous individual flowers, which cannot self-pollinate, but the possibility of fertilization by other flowers on the same plant renders them self-compatible (Klemow *et al*., 2002). Although *E. plantagineum* is self-compatible, outcrossing does occur via insect pollination, and outcrossing rates are generally high (Burdon and Brown, 1986; Burdon *et al*., 1988). The breeding system of *E. vulgare* is similar to that of *E. plantagineum* but exhibits slightly less incidence of outcrossing (Rademaker *et al*., 1999; Klemow *et al*., 2002) and predictably less genetic diversity in Australia, although other factors, such as a smaller available niche, are crucial when considering observed differences in distribution (Zhu X, Skoneczny D, Gopurenko D, Meyer L, Lepschi BJ, Weston PA, Callaway RM, Gurr GM, Weston LA. A tale of two plant invaders: comparison of the ecology and genetics of *Echium plantagineum* and *E. vulgare* in southern Australia. *Scientific Reports*, in preparation).

### Diverse population genetics and invasion history

Assessment of genetic diversity can assist in pinpointing the origins, introduction history and invasion path of a particular species, and also point out invasion-prompting factors (Burrell *et al*., 2015). Little or no genetic variation has been noted in some invasive plant populations, including barbed goat grass (*Aegilops triuncialis*; Meimberg *et al*., 2006), cat's claw creeper (*Macfadyena unguis-cati*; Prentis *et al*., 2009), North American populations of perennial pepper weed (*Lepidium latifolium*; Gaskin *et al*., 2013) and giant reed (*Arundo donax*; Ahmad *et al*., 2008). Likewise, *M. unguis-cati* showed 27 chloroplast DNA haplotypes in its native range and only one haplotype in its invaded range (Sexton *et al*., 2002; Prentis *et al*., 2009). Sometimes invaded populations are far less diverse than their source populations, and such is the case in *C. lanatus* and *C. myriocarpus* in Australia (Shaik *et al*., 2015). In other cases, the level of genetic diversity in the non-native range can be similar to the native range, as is the case in Australian *E. plantagineum* when observed using isozyme marker studies (Burdon and Brown, 1986).

Alternatively, plant invaders can exhibit post-invasion genetic diversity (Jakobs *et al*., 2004) through mutation and novel chromosomal or ploidy changes, and also by hybridization and/or introgression with closely related congeners present in the invasive range (Prentis *et al*., 2009; Meyerson and Cronin, 2013; Ndlovu *et al*., 2013). The adaptability of such species can also be influenced by post-introduction genetic changes, including adaptive evolution through selection and genetic drift, resulting in the development of locally adapted ecotypes (Hahn *et al*., 2012; Wang *et al*., 2012; Oduor *et al*., 2016). For example, genes involved in stress responses were found to be over-expressed in annual ragweed (*Ambrosia artemisiifolia*) in its introduced range (Prentis and Pavasovic, 2013). Conversely, the genetically depauperate invasive populations of Japanese knotweed (*Fallopia japonica*) showed higher epigenetic variation (leading to phenotypic variation) than genetic variation (Richards *et al*., 2012). This demonstrates that a high level of genetic diversity in the invaded population is not always an essential prerequisite to invasion success.

It is also important to evaluate the invasive population's genetic make-up at both its native location and the invaded range, as careful study can provide information on the evolutionary processes that have occurred, as well as their role in invasion success (Hornoy *et al*., 2013), and invasion history (Le Roux *et al*., 2011), including the potential number of introductions (Meimberg *et al*., 2006). Greater knowledge can also assist in the reconstruction of introduction pathways (Novak and Mack, 2001; Le Roux *et al*., 2011; Hornoy *et al*., 2013; Kelager *et al*., 2013). Multiple introductions or a single introduction of multiple genotypes of a particular species to a location from diverse source populations can also result in enhanced genetic diversity in the invaded range, e.g. rugosa rose (*Rosa rugosa*) populations were diverse in the introduced European range, suggesting multiple introductions from their source populations in Japan (Simberloff, 2009; Hornoy *et al*., 2013; Kelager *et al*., 2013). In turn, this may result in the development of locally adapted ecotypes/genotypes through natural selection (Sexton *et al*., 2002; Prentis *et al*., 2008). Additionally, potential source populations of the invader can be identified (Clark *et al*., 2013; Kelager *et al*., 2013), and these populations may help further to locate associated natural enemies (Ellstrand and Schierenbeck, 2000; Ndlovu *et al*., 2013), which may later be useful as biological control agents (Goolsby *et al*., 2006).

### Diverse management opportunities

Intraspecific diversity of an invasive plant species can have important implications for management; the genetic diversity among populations may be sufficiently great to warrant different control strategies. For example, in any invasive population, the presence of a mixture of resistant and non-resistant genotypes potentially impedes chemical and biological control (Burdon *et al*., 1981, 1984; Prentis *et al*., 2008). Hence, a genetically variable invasive plant population may be difficult to control because of naturally variable genotypes within the introduced population or the possibility of newly emerged resistant plants as a result of ongoing natural selection (Sterling *et al*., 2004). Such diverse populations may also show variable response to control by biocontrol agents (Bruckart *et al*., 2004). Knowledge of existing genetic variability in an invasive population provides further insight into the responses of weed populations to specific management strategies (Ward *et al*., 2008). Differential responses to the same management method have been observed in genetically diverse populations (Goolsby *et al*., 2006). Therefore an understanding of genetic diversity of invasive populations may help to predict the likelihood of successful management of invasive weeds, including the use of biocontrol programmes (Burdon and Marshall, 1981; Chapman *et al*., 2004; Gaskin *et al*., 2005). Knowledge of the source population of an invasive weed may also aid in finding potential biocontrol agents in the plant's native environment (Paterson *et al*., 2009).

In the case of five invasive Australian weeds described above, genetic characterization allows managers to approach each of the species differently based on the degree of variation present. Chemical control is generally used for all of these species, except *E. vulgare* (Johnson *et al*., 2006a). However, a uniform chemical control technique is recommended for populations of the two annual melon species in Australia and is currently efficacious, possibly because these species are related and are also genetically uniform (i.e. *C. lanatus* and *C. myriocarpus*). For the other three species, control tactics should logically be designed to account for regional or local differences, especially if it is shown that responses to specific controls vary among genotypes. For example, different ecotypes of a biological agent currently being tested for use against knotweed (*Fallopia* spp.) in North America have been shown to favour particular *Fallopia* species, and there are three closely related target species, including *Fallopia × bohemica*, which form a hybrid swarm (Grevstad *et al*., 2013; Clements *et al*., 2016). In *E. plantagineum*, the success of biocontrol agents in Australia was clearly associated with long-term regional adaptation of each biocontrol organism; however, genetic differences among regional plant populations may also influence biocontrol (Weston *et al*., 2012), but this requires further investigation.

## Conclusions

Comparisons among the five taxa evaluated in these case studies (Table 1) reveal a variety of patterns in species and population genetic diversity, dependent on invasion and life history and breeding systems, with implications for strategic management approaches. Invasive plants with varying levels of genetic diversity can provide important models with which to study plant invasion success. DNA sequencing technologies provide precise and clear information related to the identity of invasive plant species, along with information on genetic diversity and phylogeographic history. New sequencing technologies are also likely to continue to allow greater resolution of genetic relationships among invasive plant populations, thus improving our understanding of mechanisms driving successful invasion.
